# Supplementary material for: Targeting Hydroxybenzoic Acids to Mitochondria as a Strategy to Delay Skin Ageing: An In Vitro Approach
Source: Molecules. 2022 Sep 21;27(19):6183. doi: 10.3390/molecules27196183 (PMC9571003; doi:10.3390/molecules27196183)
Supplement: Supplementary file 1 [file molecules-27-06183-s001.zip › molecules-1883296-supplementary.pdf]

Targeting Hydroxybenzoic Acids to Mitochondria as a Strategy to Delay Skin Ageing: An In Vitro Approach

Carlos Fernandes[1,2*¥], Fernando Cagide[2¥], Jorge Simões[1], Carlos Pita[1], Eurico Pereira[1], Afonso J. C. Videira[1], Pedro Soares[2], José F. S. Duarte[1], António M. S. Santos[1], Paulo J. Oliveira[3], Fernanda Borges[2], Filomena S. G. Silva[1*]

Affiliations

[1]Mitotag, Biocant Park, Parque Tecnológico de Cantanhede, Núcleo 04, Lote 4, 3060-197 Cantanhede, Portugal

[2]CIQUP-IMS/Department of Chemistry and Biochemistry, Faculty of Sciences, University of Porto, 4169-007 Porto, Portugal

[3]CNC-Center for Neuroscience and Cell Biology, CIBB - Centre for Innovative Biomedicine and Biotechnology, University of Coimbra, 3004-504 Coimbra, Portugal

¥ These authors contributed equally to this work.

Corresponding authors:

filomenasilva@mitotag.com

carlos.fernandes@fc.up.pt

*These authors contributed equally to this work.

Supplementary Materials

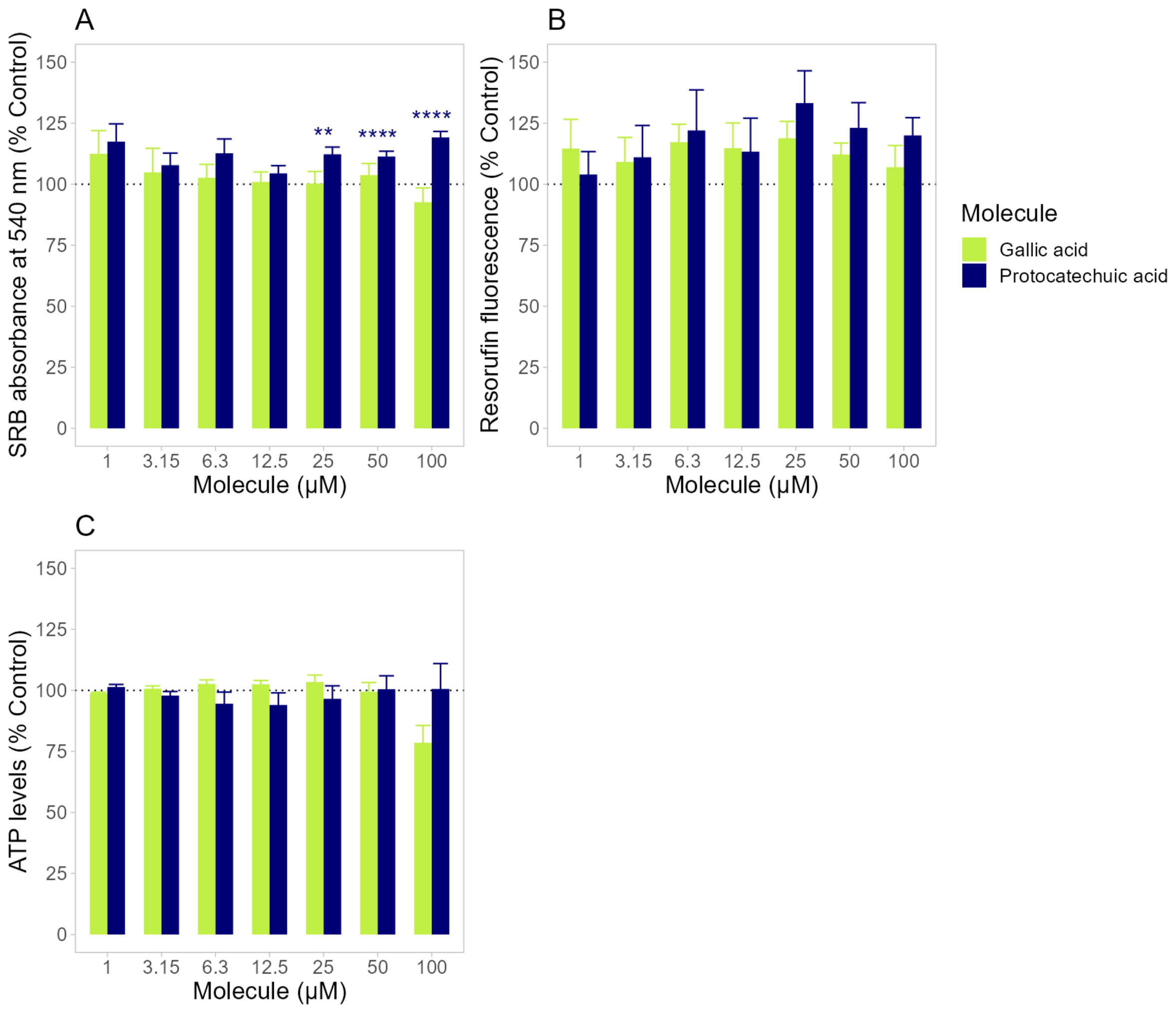

Figure S1. Effects of natural antioxidants protocatechuic and gallic acids on cell mass (A), metabolic activity (B), and intracellular ATP (C) of normal human dermal fibroblasts (NHDF). Normal human dermal fibroblasts were treated with increasing concentrations of the different molecules for 48 h. Cellular mass, metabolic activity, and ATP levels were evaluated using sulforhodamine B (SRB) assay, resazurin reduction assay, and CellTiter-Glo Luminescent Cell Viability Assay, respectively. Data are the mean ± SE of four independent experiments. The results are expressed as a percentage of the control. Statistically significant differences between control (CTL) and treated groups were evaluated using a t-test.. **** $p < 0.0001$ and ** $p < 0.01$ compared to the respective control (CTL, vehicle-treated cells), with the colours blue and green corresponding to protocatechuic and gallic acids, respectively.

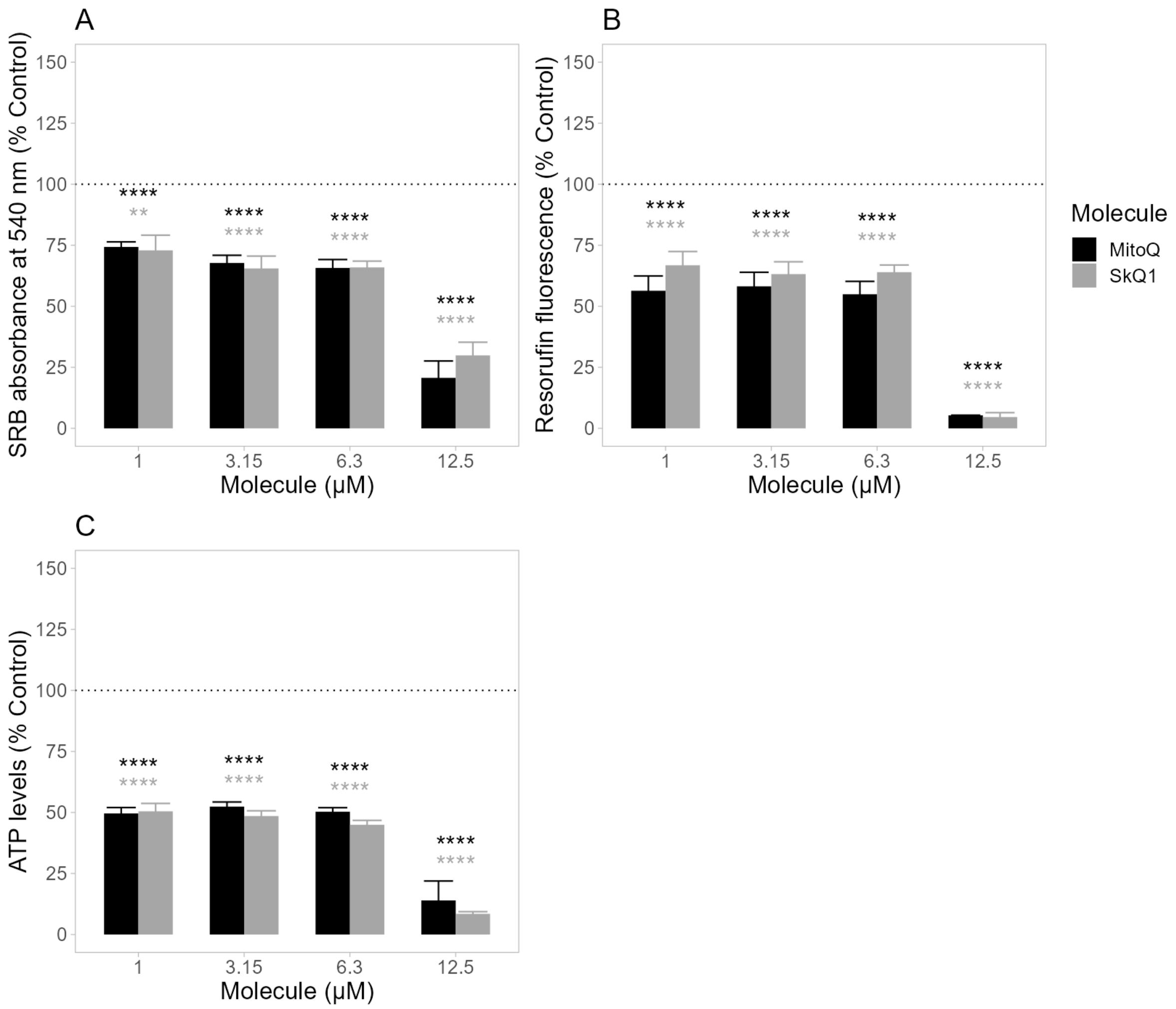

Figure S2. Effects of other mitochondrial target antioxidants MitoQ and SkQ1 on cell mass (A), metabolic activity (B), and intracellular ATP (C) of normal human dermal fibroblasts (NHDF). Normal human dermal fibroblasts were treated with increasing concentrations of the different molecules for 48 h. Cellular mass, metabolic activity, and ATP levels were evaluated using sulforhodamine B (SRB) assay, resazurin reduction assay, and CellTiter-Glo Luminescent Cell Viability Assay, respectively. Data are the mean ± SE of four independent experiments. The results are expressed as a percentage of the control. Statistically significant differences between control (CTL) and treated groups were evaluated using a t-test.. **** $p < 0.0001$ and ** $p < 0.01$ compared to the respective control (CTL, vehicle-treated cells), with the colours black and grey corresponding to MitoQ and SkQ1, respectively.

Table S1. Cytotoxicity evaluation of MB2 was performed in the *S. typhimurium* TA100 strain by the direct incorporation procedure and without metabolic activation (S9) using 5 concentrations based on the solubility profile of the test item which ranged from 0.06 up to 5 mg/plate.

amount/plate	revertants/plate			**mean**	SD	**R**
-	86.0	98	82	**88.7**	8.3	**-**
5	0	0	0	**0.0**	**0.0**	**0.0**
1.67	45	87	87	**73.0**	24.2	**0.8**
0.56	80	81	73	**78.0**	24.4	**0.9**
0.19	58	90	63	**70.3**	17.2	**0.8**
0.06	65	72	64	**67.0**	4.4	**0.8**

Average plate counts were presented with the mean and the standard deviation for each set of triplicates per test item concentration and was used to calculate the ratio of colonies per exposed plate compared to the corresponding negative control.

Table S2. Cytotoxicity evaluation of the MB2 (0.02-1.67 mg/plate) was performed in the *S. typhimurium* (TA98 and TA100 strains), E. Coli (WP2 strain) and *S. typhimurium* (TA1535 and TA1537 strains) by the direct incorporation procedure and without metabolic activation.

								**TA98**
		**Amount/plate**	**Revertants/plate**			**Mean**	**SD**	**R**
**Solvent:**	**DMSO**	-	**14**	**29**	**19**	**20.7**	**7.6**	-
**Reference item (μg):**	**2-nitrofluorene**	**5.0**	**624**	**738**	**820**	**727.3**	**98.4**	**35.2**
	**Test item mg**	**1.67**	**19**	**18**	**15**	**17.3**	**2.1**	**0.8**
		**0.56**	**25**	**25**	**27**	**25.7**	**1.2**	**1.2**
		**0.19**	**20**	**20**	**16**	**18.7**	**2.3**	**0.9**
		**0.06**	**23**	**31**	**19**	**24.3**	**6.1**	**1.2**
		**0.02**	**25**	**17**	**22**	**21.3**	**4.0**	**1.0**
		**Amount/plate**	**Revertants/plate**			**Mean**	**SD**	**TA100**
**Solvent:**	**DMSO**	-	**105**	**92**	**92**	**96.3**	**7.5**	-
**Reference item (μg):**	**Sodium azide**	**2.5**	**846**	**881**	**719**	**815.3**	**85.2**	**8.5**
	**Test item mg**	**1.67**	**76**	**108**	**87**	**90.3**	**16.3**	**0.9**
		**0.56**	**80**	**100**	**94**	**91.3**	**10.3**	**0.9**
		**0.19**	**77**	**106**	**108**	**97.0**	**17.3**	**1.0**
		**0.06**	**63**	**91**	**92**	**82.0**	**16.5**	**0.9**
		**0.02**	**65**	**76**	**89**	**76.7**	**12.0**	**0.8**

		Amount/plate	Revertants/plate			Mean	SD	TA1535
Solvent:	DMSO	-	15	18	14	15.7	2.1	-
Reference item (µg):	Sodium azide	3.5	984	1118	1033	1045.0	67.8	66.7
	Test item mg	1.67	14	13	14	13.7	0.6	0.9
		0.56	14	13	19	15.3	3.2	1.0
		0.19	13	17	16	15.3	2.1	1.0
		0.06	12	16	13	13.7	2.1	0.9
		0.02	22	21	13	18.7	4.9	1.2

		Amount/plate	Revertants/plate			Mean	SD	TA1537
Solvent:	DMSO	-	10	7	5	7.3	2.5	-
Reference item (µg):	9-aminoacridine	45.0	153	183	203	179.7	25.2	24.5
	Test item mg	1.67	5	6	4	5.0	1.0	0.7
		0.56	6	4	7	5.7	1.5	0.8
		0.19	4	5	7	5.3	1.5	0.7
		0.06	8	4	7	6.3	2.1	0.9
		0.02	7	6	2	5.0	2.6	0.7

		Amount/plate	Revertants/plate			Mean	SD	WP2
Solvent:	DMSO	-	284	333	225	280.7	54.1	-
Reference item (µg):	4-nitroquinoline-N-oxide	0.4	1702	2002	1914	1872.7	154.2	6.7
	Test item mg	1.67	187	226	174	195.7	27.1	0.7
		0.56	215	284	228	242.3	36.7	0.9
		0.19	249	276	219	248.0	28.5	0.9
		0.06	262	299	261	274.0	21.7	1.0
		0.02	211	259	214	228.0	26.9	0.8

Average plate counts were presented with the mean and the standard deviation for each set of triplicates per test item concentration and was used to calculate the ratio of colonies per exposed plate (R) compared to the corresponding negative control.

Table S3. Cytotoxicity evaluation of the MB2 (0.02-1.67 mg/plate) was performed in the *S. typhimurium* (TA98 and TA100 strains), E. Coli (WP2 strain) and *S. typhimurium* (TA1535 and TA1537 strains) by the pre-incubation procedure and without metabolic activation.

								TA98
		Amount/plate	Revertants/plate			Mean	SD	R
Solvent:	DMSO	-	19	15	33	22.3	9.5	-
Reference item (µg):	2-nitrofluorene	5.0	511	543	539	531.0	17.4	23.8
	Test item mg	1.67	3	5	2	3.3	1.5	0.1
		0.56	15	15	22	17.3	4.0	0.8
		0.19	18	15	13	15.3	2.5	0.7
		0.06	12	14	15	13.7	1.5	0.6
		0.02	15	19	22	18.7	3.5	0.8

		Amount/plate	Revertants/plate			Mean	SD	TA100
Solvent:	DMSO	-	73	94	79	82.0	10.8	-
Reference item (µg):	Sodium azide	2.5	728	885	667	760.0	112.5	9.3
	Test item mg	1.67	97	99	106	100.7	4.7	1.2
		0.56	67	83	59	69.7	12.2	0.8
		0.19	66	76	60	67.3	8.1	0.8
		0.06	70	84	59	71.0	12.5	0.9
		0.02	87	78	62	75.7	12.7	0.9

		Amount/plate	Revertants/plate			Mean	SD	TA1535
Solvent:	DMSO	-	13	10	14	12.3	2.1	-
Reference item (µg):	Sodium azide	3.5	1156	1129	915	1066.7	132.0	86.5
	Test item mg	1.67	10	11	10	10.3	0.6	0.8
		0.56	17	14	32	21.0	9.6	1.7
		0.19	14	9	14	12.3	2.9	1.0
		0.06	14	15	10	13.0	2.6	1.1
		0.02	16	20	14	16.7	3.1	1.4

		Amount/plate	Revertants/plate			Mean	SD	TA1537
Solvent:	DMSO	-	4	9	10	7.7	3.2	-
Reference item (µg):	9-aminoacridine	45.0	176	143	211	176.7	34.0	23.0

	Test item mg	1.67	8	14	7	9.7	3.8	1.3
		0.56	7	6	4	5.7	1.5	0.7
		0.19	7	6	6	6.3	0.6	0.8
		0.06	2	3	10	5.0	4.4	0.7
		0.02	13	4	7	8.0	4.6	1.0
		Amount/plate	Revertants/plate			Mean	SD	WP2
Solvent:	DMSO	-	296	295	270	287.0	14.7	-
Reference item (µg):	4-nitroquinoline-N-oxide	0.4	2270	2334	2025	2209.7	163.1	7.7
	Test item mg	1.67	141	156	128	141.7	14.0	0.5
		0.56	334	257	236	275.7	51.6	1.0
		0.19	365	343	260	322.7	55.4	1.1
		0.06	320	291	256	289.0	32.0	1.0
		0.02	259	279	195	244.3	43.9	0.9

Average plate counts were presented with the mean and the standard deviation for each set of triplicates per test item concentration and was used to calculate the ratio of colonies per exposed plate (R) compared to the corresponding negative control.

Table S4. Cytotoxicity evaluation of the MB2 (0.02-1.67 mg/plate) was performed in the *S. typhimurium* (TA98 and TA100 strains), E. Coli (WP2 strain) and *S. typhimurium* (TA1535 and TA1537 strains) by the direct incorporate procedure and with metabolic activation.

								TA98
		Amount/plate	Revertants/plate			Mean	SD	R
Solvent:	DMSO	-	17	22	19	19.3	2.5	-
Reference item (µg):	2-amino-anthracene	1.5	643	620	458	573.7	100.8	29.7
	Test item mg	1.67	14	23	21	19.3	4.7	1.0
		0.56	16	31	24	23.7	7.5	1.2
		0.19	23	27	16	22.0	5.6	1.1
		0.06	12	24	19	18.3	6.0	0.9
		0.02	15	18	23	18.7	4.0	1.0
		Amount/plate	Revertants/plate			Mean	SD	TA100
Solvent:	DMSO	-	98	113	79	96.7	17.0	-
Reference item (µg):	2-amino-anthracene	2.5	1865	1824	2104	1931.0	151.2	20.0

	Test item mg	1.67	78	88	74	80.0	7.2	0.8
		0.56	73	97	69	79.7	15.1	0.8
		0.19	60	91	76	75.7	15.5	0.8
		0.06	68	101	77	82.0	17.1	0.8
		0.02	82	97	88	89.0	7.5	0.9
		Amount/plate	Revertants/plate			Mean	SD	TA1535
Solvent:	DMSO	-	12	15	15	14.0	1.7	-
Reference item (µg):	2-amino-anthracene	30	425	328	393	382.0	49.4	27.3
	Test item mg	1.67	11	19	9	13.0	5.3	0.9
		0.56	13	18	14	15.0	2.6	1.1
		0.19	13	19	14	15.3	3.2	1.1
		0.06	14	12	18	14.7	3.1	1.0
		0.02	12	15	10	12.3	2.5	0.9
		Amount/plate	Revertants/plate			Mean	SD	TA1537
Solvent:	DMSO	-	7	8	5	6.7	1.5	-
Reference item (µg):	2-amino-anthracene	2.5	200	172	169	180.3	17.1	27.1
	Test item mg	1.67	9	11	6	8.7	2.5	1.3
		0.56	12	10	7	9.7	2.5	1.5
		0.19	9	7	7	7.7	1.2	1.2
		0.06	4	9	8	7.0	2.6	1.1
		0.02	7	8	4	6.3	2.1	1.0
		Amount/plate	Revertants/plate			Mean	SD	WP2
Solvent:	DMSO	-	266	230	267	254.3	21.1	-
Reference item (µg):	2-amino-anthracene	30	2002	2123	2064	2063.0	60.5	8.1
	Test item mg	1.67	245	235	232	237.3	6.8	0.9
		0.56	335	326	245	302.0	49.6	1.2
		0.19	318	365	284	322.3	40.7	1.3
		0.06	359	336	283	326.0	39.0	1.3
		0.02	315	328	268	303.7	31.6	1.2

Average plate counts were presented with the mean and the standard deviation for each set of triplicates per test item concentration and was used to calculate the ratio of colonies per exposed plate compared to the corresponding negative control.

Table S5. Cytotoxicity evaluation of the MB2 (0.02-1.67 mg/plate) was performed in the *S. typhimurium* (TA98 and TA100 strains), E. Coli (WP2 strain) and *S. typhimurium* (TA1535 and TA1537 strains) by the pre-incubation procedure and with metabolic activation (S9).

								TA98
		Amount/plate	Revertants/plate			Mean	SD	R
Solvent:	DMSO	-	28	18	24	23.3	5.0	-
Reference item (µg):	2-amino-anthracene	1.5	551	588	424	521.0	86.0	22.3
	Test item mg	1.67	18	24	22	21.3	3.1	0.9
		0.56	23	17	11	17.0	6.0	0.7
		0.19	31	27	18	25.3	6.7	1.1
		0.06	24	21	27	24.0	3.0	1.0
		0.02	31	23	29	27.7	4.2	1.2
		Amount/plate	Revertants/plate			Mean	SD	TA100
Solvent:	DMSO	-	132	110	90	110.7	21.0	-
Reference item (µg):	2-amino-anthracene	2.5	1040	1374	1137	1183.7	171.8	10.7
	Test item mg	1.67	78	87	66	75.3	8.3	0.7
		0.56	110	97	98	101.7	7.2	0.9
		0.19	97	132	103	110.7	18.7	1.0
		0.06	103	98	99	100.9	2.6	0.9
		0.02	96	119	107	107.3	11.5	1.0
		Amount/plate	Revertants/plate			Mean	SD	TA1535
Solvent:	DMSO	-	34	18	19	23.7	9.0	-
Reference item (µg):	2-amino-anthracene	30	509	437	332	426.0	89.0	18.0
	Test item mg	1.67	8	15	8	10.3	4.0	0.4
		0.56	20	21	17	19.3	2.1	0.8
		0.19	14	23	21	19.3	4.7	0.8
		0.06	22	21	11	18.0	6.1	0.8
		0.02	18	31	16	21.7	8.1	0.9
		Amount/plate	Revertants/plate			Mean	SD	TA1537
Solvent:	DMSO	-	7	10	7	8.0	1.7	-

Reference item (µg):	2-amino-anthracene	2.5	127	107	118	117.3	10.0	14.7
	Test item mg	1.67	7	9	9	8.3	1.2	1.0
		0.56	6	7	7	6.7	0.6	0.8
		0.19	11	8	7	8.7	2.1	1.1
		0.06	8	7	6	7.0	1.0	0.9
		0.02	9	5	9	7.7	2.3	1.0
		Amount/plate	Revertants/plate			Mean	SD	WP2
Solvent:	DMSO	-	321	337	276	311.3	31.6	-
Reference item (µg):	2-amino-anthracene	30	2315	2156	1987	2152.7	164.0	6.9
	Test item mg	1.67	247	216	185	216.0	31.0	0.7
		0.56	220	256	182	219.3	37.0	0.7
		0.19	271	261	187	239.7	45.9	0.8
		0.06	237	266	191	231.3	37.8	0.7
		0.02	222	253	171	215.3	41.4	0.7

Average plate counts were presented with the mean and the standard deviation for each set of triplicates per test item concentration and was used to calculate the ratio of colonies per exposed plate compared to the corresponding negative control.

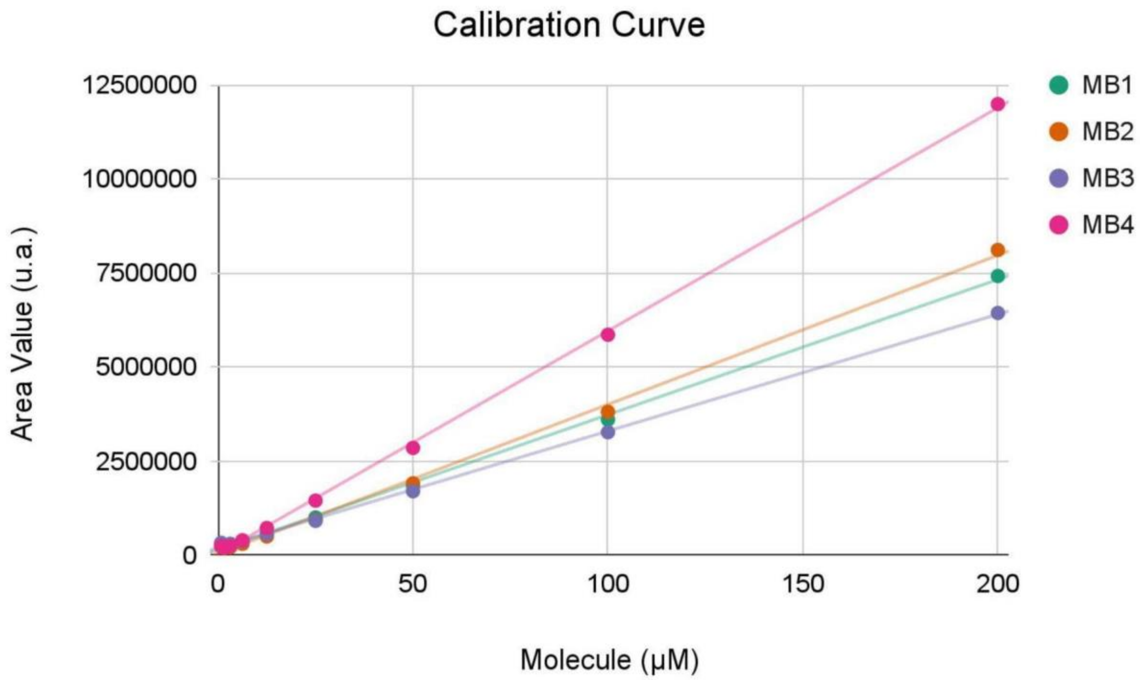

Concentration (µM)	MB1	MB2	MB3	MB4
0.8	274533	233138	327994	240883
1.6	236359	190234	261272	200359
3.1	275956	210666	301910	243725
6.3	364599	301142	376904	396598
12.5	577174	497570	565023	722481
25.0	1001997	927611	912839	1450640
50.0	1845605	1910422	1700396	2852283
100.0	3602164	3810442	3269178	5861325
200.0	7422287	8115240	6439844	11994915
R-Squared	0.999	0.998	0.999	0.999

Figure S3. Calibration curves of MitoBENs. Represents the linear regression curves of crescent concentrations of MB1-4 with the respective areas of each concentration injected in the UHPLC used to determine the concentrations at the different time-points.
